# Complete genome sequence of *Mycoplasmopsis synoviae* strain CQTL01 derived from the knee joint of a broiler

**DOI:** 10.1128/mra.00461-26

**Published:** 2026-06-30

**Authors:** Xiuwu Lian, Yunchong Ma, Honglei Ding

**Affiliations:** 1 Laboratory of Veterinary Mycoplasmology, College of Veterinary Medicine, Southwest University 359443, El Paso, Texas, USA; University of Maryland Baltimore, Baltimore, Maryland, USA

**Keywords:** *Mycoplasmopsis synoviae*, infectious synovitis, broiler, knee joint

## Abstract

We present the complete genome of *Mycoplasmopsis synoviae* strain CQTL01, which was isolated from the knee joint of a broiler. The genome is 790,691 bp long, with a GC content of 28.39%. NCBI PGAP v6.11 identified 670 protein-coding genes.

## ANNOUNCEMENT

*Mycoplasmopsis synoviae* is a tiny, fastidious, cell wall-free pathogenic bacterium that causes substantial economic losses to the poultry industry (1). This pathogen primarily induces acute or chronic infectious synovitis, air sacculitis, and growth retardation in broilers. In commercial laying hens, key clinical consequences are reduced egg production and hatchability, as well as eggshell apex abnormalities (2). Strain CQTL01 was isolated from a broiler farm in Tongliang District, Chongqing Municipality, China, on 19 March 2022 (3).

Knee joint with infectious synovitis obtained from broiler was rinsed and surface-disinfected with 70% (vol/vol) ethanol. Inflammatory exudate was inoculated into KM2 complete broth medium (3) and incubated at 37°C until color change. After four passages, cultures were streaked on KM2 agar plates and incubated at 37°C until colony formation was visualized via optical microscopy. Typical fried-egg-shaped colonies were picked up and further propagated in KM2 complete broth. Before genome sequencing, the isolate was identified by qPCR according to NY/T 4035-2021 (4). Genomic DNA was extracted using the TIANamp Bacteria DNA Kit (TIANGEN, China) and quality-checked by gel electrophoresis and spectrophotometry.

For sequencing on the DNBSEQ platform, 1 µg of DNA was randomly fragmented using a Covaris instrument, and 300–400 bp fragments were size-selected with the Agencourt AMPure XP-Medium kit to construct single-strand circular DNA libraries using the MGIEasy Universal DNA Library Prep Set (MGI-Shenzhen, China). Qualified libraries were sequenced on a BGISEQ-500 sequencer with paired-end 2 × 150 bp reads (5). A total of 1.314 Gb of raw data (8,764,210 reads) were generated; SOAPnuke v1.5.5 (6) was used to remove reads with ≥40% low-quality bases (*Q* ≤ 20), ≥40% Ns, adapter contamination, and duplicate reads, and 1.291 Gb of clean data were retained, corresponding to ~1,632 × coverage.

On the PacBio platform, DNA was sheared to 10–15 kb using a g-TUBE, and the ends of the DNA fragments were ligated to form dumbbell structures using the SMRTbell Template Prep Kit v1.0 (Pacific Biosciences). PacBio subreads shorter than 1 kb were discarded. PacBio sequencing yielded 1,272,083 filtered subreads totaling 12,598,473,444 bp (mean length: 9,903 bp; N50: 10,491 bp; N90: 6,853 bp; maximum length: 238,823 bp), providing ~15,932× coverage. Subread correction and genome assembly were performed using both Canu v1.5 (parameters: estn = 24, npruseGrid = 0, corOvlMemory = 4) and Falcon v0.3.0 (parameters: -v -dal8 -t32 -h60 -e.96 -l500 -s100 -H3000) software, with the optimal assembly selected (7, 8). The assembly was polished for single-base error correction using DNBSEQ data with GATK v3.4-0-g7e26428 (parameters: -cluster 2 -window 5 -stand_call_conf 50 -stand_emit_conf 10.0 -dcov 200 MQ0 ≥ 4) (9) and was subsequently circularized with Circlator v1.5.5 (10). Genome annotation was performed using the NCBI Prokaryotic Genome Annotation Pipeline (PGAP) v6.11 (11).

The *M. synoviae* CQTL01 genome is 790,691 bp in length with a GC content of 28.39%. Genome annotation through NCBI PGAP v6.11 identified 670 protein-coding genes, 44 RNA genes, and 18 pseudogenes. The RNA genes include 34 tRNAs, 7 rRNAs, and 3 ncRNAs.

## Data Availability

The complete genome sequence of *Mycoplasmopsis synoviae* strain CQTL01 has been deposited in GenBank under accession number CM160071.1, BioProject number PRJNA1393869, and BioSample number SAMN54314994. The raw sequence reads are available under Sequence Read Archive accession numbers SRR36603514 (PacBio) and SRR36721229 (BGISEQ).
